# Phenolic Fractions from Walnut Milk Residue: Antioxidant Activity and Cytotoxic Potential

**DOI:** 10.3390/plants13243473

**Published:** 2024-12-11

**Authors:** Pamela Ruth Toledo-Merma, María Fernanda Arias-Santé, Miguel Ángel Rincón-Cervera, Omar Porras, Raquel Bridi, Samantha Rhein, Martina Sánchez-Contreras, Paulina Hernandez-Pino, Nicolás Tobar, Luis Puente-Díaz, Adriano Costa de Camargo

**Affiliations:** 1 Department of Food Science and Chemical Technology, Faculty of Chemical and Pharmaceutical Sciences, University of Chile, Av. Doctor Carlos Lorca 964, Independencia, Santiago 8380494, Chile; pamela.toledo@ug.uchile.cl (P.R.T.-M.); lpuente@ciq.uchile.cl (L.P.-D.); 2 Institute of Nutrition and Food Technology, University of Chile, El Líbano 5524, Macul, Santiago 7830490, Chile; ma.fernanda.arias@inta.uchile.cl (M.F.A.-S.); marincer@inta.uchile.cl (M.Á.R.-C.); omar.porras@inta.uchile.cl (O.P.); samy.rhein@gmail.com (S.R.); martina.sanchez@ug.uchile.cl (M.S.-C.); paulina.hernandez@inta.uchile.cl (P.H.-P.); ntobar@inta.uchile.cl (N.T.); 3 Department of Agronomy, Food Technology Division, University of Almería, 04120 Almería, Spain; 4 Department of Pharmacological and Toxicological Chemistry, Faculty of Chemical and Pharmaceutical Sciences, University of Chile, Av. Doctor Carlos Lorca 964, Independencia, Santiago 8380000, Chile; raquelbridi@ciq.uchile.cl; 5 Department of Agricultural, Food and Nutritional Science, University of Alberta, Edmonton, AB T6G 2P5, Canada

**Keywords:** *Juglans regia* L., walnut residues, soluble phenolics, insoluble-bound phenolic hydrolysates, natural antioxidants, Caco-2 cells

## Abstract

Walnut milk residues (WMR) were investigated for the first time through their phenolic characterization including soluble (free, esterified, and etherified) phenolics and those released from their insoluble-bound form (insoluble-bound phenolic hydrolysates, IBPHs) and their antioxidant properties. Free phenolics were recovered and alkaline or acid hydrolysis were used to recover the remaining phenolic fractions. Total phenolic compounds (TPCs) and their antioxidant activity were analyzed by Folin–Ciocalteu, FRAP, and ORAC methods, respectively. Soluble phenolics (free + esterified + etherified fractions) showed a higher TPC (275.3 mg GAE 100 g^−1^ dw) and antioxidant activity (FRAP: 138.13 µmol TE g^−1^ dw; ORAC: 45.41 µmol TE g^−1^ dw) with respect to the IBPH. There was a significant correlation between TPC and FRAP and ORAC values regardless of the fraction and tested sample. Phenolic acids and flavonoids were identified and quantified by ultra-performance liquid chromatography–electrospray tandem mass spectrometry (UPLC-ESI-MS/MS). Gallic acid, mainly in the free form (3061.0 µg 100 g^−1^), was the most representative, followed by biochanin A, identified for the first time in a walnut product and mostly present in the fraction released from the esterified form (593.75 µg 100 g^−1^). No detrimental cytotoxic impact on Caco-2 cells was observed. Hence, WMR could be considered a potential source for the development of nutraceutical and/or antioxidant food additives.

## 1. Introduction

Walnut (*Juglans regia* L.), an edible nut of high nutritional value, is mainly composed of unsaturated fatty acids, proteins, dietary fiber, minerals, and B-complex vitamins, in addition to vitamin E (tocopherols) and phytosterols [1,2,3,4]. In addition, walnuts contain significant amounts of compounds of a phenolic nature [5,6]. These compounds have shown potential beneficial effects on human health against chronic non-communicable diseases mediated by oxidative stress (e.g., some types of cancer, type II diabetes, and cardiovascular diseases) and neurodegenerative diseases due to their antioxidant and anti-inflammatory capacity in in vitro and in vivo studies [7,8,9,10].

Phenolic compounds are found in different forms depending on their association with the food matrix. There are free, esterified, and etherified soluble phenolics as well as insoluble-bound phenolics [11,12]. However, most of the reported studies only consider the phenolic analysis and antioxidant activity of the soluble phenolic fraction, generally evaluated after obtaining a crude extract, omitting the contribution of each of the phenolic fractions, including free, esterified, etherified soluble, and insoluble-bound phenolics [13,14]. The free soluble phenolics are found within the vacuoles of the vegetal cell. The insoluble-bound phenolics are bound to carbohydrates and proteins of the plant matrix cell wall by covalent bonds, which can be released by alkaline hydrolysis [15].

The antioxidant potential of walnut has been evidenced by different studies [3,16,17]. Likewise, some walnut agroindustrial by-products such as the outer green husk, hard brown shell, leaves, and flowers of the walnut tree, as well as other walnut residues, have been shown to possess high antioxidant capacity, reducing power, and/or immunomodulatory activity [12,18,19]. The phenolic profile was also addressed, but none of these studies focused on free, esterified, etherified, and/or phenolics resealed from the insoluble-bound form or evaluated the antioxidant properties of IBPH. The plant-based beverages industry is expected to grow at an annual rate of 12.35% globally between 2023 and 2030 [20]. Therefore, the generation of other by-products from the walnut industry, such as walnut milk residues (WMR), is also expected to grow. In addition, by 2028, total consumption of these products is expected to exceed 437 million liters [21]. This consumption includes plant-based beverages produced at large scale as well as homemade options. WMR has not widely being reused in the plant-based beverage processing industry, probably due to the lack of suitable processing technologies and the costs involved.

Defatted walnut residue (DWR) was shown to be a rich source of phenolic compounds with antioxidant potential [22]. However, no previous studies have been reported on the phenolic characterization and antioxidant activity from the different fractions of soluble (free, esterified, and etherified) phenolics and IBPH from WMR, nor the percentage of phenolic compounds in the raw material that would be discarded by not considering WMR as a source of phenolic compounds. Furthermore, the study of this by-product is important in the context of waste reduction, optimization of natural resources, and the potential economic and health benefits of the bioactive compounds present in this residue, all of which results in an improvement in the environmental impact and suggest its possible use as a food ingredient or additive.

Considering the multiple applications that have been given to soy milk residue, known as okara, WMR may also be reused as a flour-type food ingredient to increase the fiber and protein content of different food products. For example, it could be added to noodles, bread, cakes, and cookies, to name a few. In addition, WMR may be used for the development of edible films, culture medium, or dietary supplements [23,24]. However, assessing the cytotoxic potential of phenolic extracts is essential to anticipate the safety of these compounds before their application in products for human consumption [25]. The Caco-2 cell line distinguishes itself from other human-derived cell lines because it represents a key site of interaction between digestion-derived compounds and cells responsible for absorbing bioactive substances with potential human applications [26].

Considering the literature gap, the novel information provided here may not only impact the food industry but also the agricultural and nutraceutical industry. Therefore, the objective of the present study was to characterize the soluble (free, esterified, and etherified) phenolics and IBPH fractions of WMR for their phenolic content and antioxidant and cytotoxic potential, as well as to characterize the phenolics by ultra-performance liquid chromatography–electrospray tandem mass spectrometry (UPLC-ESI-MS/MS).

## 2. Results and Discussion

### 2.1. Total Phenolic Content

Walnuts contain different phenolic compounds, mainly phenolic acids and flavonoids [27]. In this study, the TPCs of each of the fractions, both soluble (free, esterified, and etherified) and IBPH, were determined in the extracts from DWR and WMR, including extraction yield (X_0_), which are shown in Table 1. Although the soluble phenol fractions of DWR had a higher representation (62.79%), it should be noted that the estimation of total phenolics would indicate that the DWR would present a significant quantity of IBPH (37.21%) that mostly tends to be more difficult to extract from the matrix since they are strongly attached to the cell wall [15]. The soluble-IBPH ratio (S/I) in DWR was 1.69. Moreover, within the soluble fractions, the free ones represented 61.02%, followed by esterified and etherified soluble ones with 22.45% and 16.53%, respectively.

Some previous studies were carried out which determined the content of total phenolics from a crude extract, that is, without considering the fractionation of phenolics as addressed in the present study. A TPC of 540.08 mg GAE 100 g^−1^ dw was reported from walnuts of the same Chandler variety used in the present study, but of USA origin [13]. In addition, Trandafir et al. [17] employed walnuts of 12 different genotypes from Romania with results of total phenolics between 1131 and 2892 mg GAE 100 g^−1^ dw. Likewise, Labuckas et al. [28] reported a TPC of 1630 mg GAE 100 g^−1^ dw from whole walnut flour making use of 60% methanol and 70% ethanol as the extraction solvent. In addition, another study where walnuts from two different growing areas in Chile (near the Andes Mountains and the Pacific Ocean) of different colors of the Chandler variety were analyzed reported total phenol contents in the range of 570 to 1270 mg GAE 100 g^−1^ dw [29]. In addition, Slatnar et al. [14] analyzed four varieties of walnuts from France and the USA where their results were in the range of 609.54 to 1092.51 mg GAE 100 g^−1^ dw.

Furthermore, very few studies have been carried out on the analysis of total phenolics from the different free, esterified, etherified, and IBPH fractions. It is important to note that the present study is the first to evaluate the total phenolic content of all four fractions of phenolic compounds from defatted walnuts. Wu et al. [5] analyzed the skinless walnut of Chinese origin, obtaining a TPC of the fractions of free and esterified soluble phenolics and IBPH of 1536, 500, and 749 mg GAE 100 g^−1^ dw, respectively. Likewise, another study was carried out using whole walnuts and obtained higher ranges of free and esterified TPC but nonetheless similar IBPH content (341–530 mg GAE 100 g^−1^ dw), since what was reported in the present study is within the range [3]. These differences in TPC could depend on several factors such as the variety and species of walnut, its origin, its quality attributes, and climatic, environmental, and agronomic factors, as well as the extraction methods used, type of solvent applied, and different conditions of preparation and integrity of the plant matrix [14,29,30,31].

Regarding WMR, the fractions of free, esterified, and etherified phenolics and IBPH represented 28.08, 17.66, 16.37, and 37.89%, respectively. The ratio of soluble phenolics to IBPH (S/I) present in this type of residue was 1.64. No previous studies have been reported on the content of total phenolics from extracts obtained from the different fractions of soluble phenolics and IBPH from WMR. In this study, it was shown that 44% w/w of the phenolics present in the walnuts were not extracted during the production of walnut milk and remained in the respective residue (WMR). Therefore, a significant percentage would be discarded by not considering WMR. There are very few studies conducted where total phenolics were analyzed from plant-based beverage residues; however, mostly they have been conducted from soybean plant-based beverage residue (also known as okara). Canaan et al. [32], reported a TPC of 298 mg GAE 100 g^−1^ dw from the crude extract of okara residue. In addition, Vital et al. [33] analyzed by the same Folin–Ciocalteu method with certain differences the residue of soybean plant-based beverage, determining a TPC of 130.50 mg GAE 100 g^−1^ dw. Therefore, taking into account that WMR presents a TPC of 443.25 mg GAE 100 g^−1^ dw from the fractions of both free, esterified, and etherified soluble phenolics and IBPH, WMR could also be considered an important source of phenolic compounds in order to be valorized by the walnut industry.

### 2.2. Antioxidant Activity

The FRAP values of the phenolic fractions of both DWR and WMR are detailed in Table 1. The mechanism of operation of the FRAP assay, by electron transfer, can be influenced by the presence, number, and configuration of the hydroxyl groups present in the phenolic compounds in the matrix. This can exert an influence on the chemical reactions that take place, which are related to the ability of phenolics to inactivate or reduce reactive oxygen species (ROS) and/or metal ions such as ferric ions in the case of the FRAP assay [34,35]. There was a significant correlation between the FRAP reducing power and TPC (r = 0.7516, *p* < 0.05). Furthermore, a positive correlation (*p* < 0.05) was found between the FRAP of the free fraction (r = 0.9955), esterified fraction (r = 0.9877), etherified fraction (r = 0.9976), and IBPH (r = 0.9970) and TPC, which demonstrates the antioxidant potential of the phenolic fractions in reducing unstable ions.

Free soluble phenolic fraction of the DWR had the highest reducing power (887.56 µmol TE g^−1^ dw) of all the phenolic fractions evaluated, including those of WMR. There was no significant difference between the reducing power of both esterified and etherified soluble phenolic fractions of the DWR representing 7.61 and 7.59% of the total, respectively. It should be noted that IBPH contributed 13.76% of the total reducing power present in the DWR. The ratio of the reducing power of soluble/insoluble phenolics (S/I) was 6; thus, soluble phenolics supply six-fold more reducing power compared to insoluble phenolics.

With regard to WMR, the free soluble phenolic fraction was also the major contributor to the reducing power of all fractions (55.9%) followed by the IBPH with approximately 30% of the total reducing power present in this residue. The etherified soluble phenolic fraction had the lowest reducing power (8.97 µmol TE g^−1^ dw), which correlated with the lowest TPC present in this fraction (72.55 mg GAE 100 g^−1^ dw).

It is important to note that there was a highly significant correlation (r = 0.9942, *p* < 0.05) between gallic acid and reducing power (FRAP), which would suggest that perhaps gallic acid would be one of the main phenolic compounds responsible for conferring this ferric to ferrous ion reducing capacity to the phenolic fractions from WMR.

The reducing power of the phenolics present in DWR was much higher than that of WMR (approximately six-fold more). However, it should be noted that this residue could also be valorized and used because it presented a significant antioxidant power (196.07 µmol TE g^−1^ dw). As a reference, according to Arranz et al. [16], they determined an antioxidant capacity of 114.92 TE g^−1^ dw from the crude extract, without phenolic fractionation, of defatted Spanish walnuts. Christopoulos and Tsantili [36] analyzed Californian walnuts grown in Central Greece of the same variety as the present study (Chandler) where they reported a reducing power of 153.40 µmol TE g^−1^ dw from a crude extract.

ORAC measurement is used in the presence of reactive oxygen species (ROS), especially peroxyl radicals, which are found in biological systems and can also be generated in lipid-rich foods during lipid oxidation. The ORAC values of all phenolic fractions from both DWR and WMR are shown in Table 1. The total oxygen radical absorbance capacity (ORAC) of WMR (66.25 µmol TE g^−1^) represented 50% of the ORAC value of the DWR (132.21 µmol TE g^−1^). In addition, there were significant differences between the free, esterified, and etherified soluble phenolics and IBPH from DWR, which differed in the case of WMR, where there was no significant difference between the free (15.52 µmol TE g^−1^) and etherified (16.05 µmol TE g^−1^) soluble phenolic fractions. In DWR, the soluble phenolic fractions (free, esterified, and etherified) accounted for 61.83% of the total ORAC value, while in WMR, the soluble phenolic fractions had a more representative ORAC value of 68.55% compared to the insoluble phenolic fraction. The ORAC value ratio of soluble over insoluble phenolics (S/I) was 1.62 and 2.18 in DWR and WMR, respectively. The ORAC values were significantly different between DWR and WMR regardless of the fraction. The ORAC value of the free phenolic fraction of WMR was 42% of that from DWR.

There was a significant positive correlation between TPC values and ORAC values (r = 0.9133, *p* < 0.05) of all extracts evaluated for both DWR and WMR. Furthermore, a positive correlation (*p* < 0.05) was found between the ORAC values of the free fraction (r = 0.9990), esterified fraction (r = 0.9976), etherified fraction (r = 0.9915), and IBPH (r = 0.9975) and TPC, demonstrating the correspondence of antioxidant potential from the content of phenolics present in each fraction in inducing absorption of peroxyl and alkoxyl radicals.

So far, no studies have analyzed the antioxidant potential (ORAC) of the free, esterified, etherified, and IBPH fractions from walnuts. However, some studies have measured the ORAC values of crude walnut extracts, including from defatted walnuts from different varieties [37]. Another study found an ORAC value of 187.18 µmol TE g^−1^ in defatted walnuts [16], which is higher than that of the present study. The differences in ORAC values may be due to factors such as walnut variety, preparation methods, extraction techniques, and the use of different solvents.

### 2.3. Identification and Quantification of Phenolic Compounds from WMR

Previous studies have succeeded in qualitatively and quantitatively characterizing the main phenolic compounds present in walnuts [5,27]. However, in view of the need to valorize the residues coming from the walnut industry, in the present study, certain phenolic compounds of mainly phenolic acids and flavonoid types were identified and quantified from the four phenolic extracts or fractions (free, esterified, etherified, and IBPH) from WMR for the first time.

Table 2 shows the phenolic compounds that were identified and quantified using UPLC-ESI-MS/MS. Ten phenolic compounds, including six phenolic acids and four flavonoids, were identified in WMR. Considering the totality of all fractions, gallic acid and biochanin A were the most abundant, with 74.26 and 16.81%, respectively, of the total phenolic compounds content. Gallic acid, a phenolic acid also known as 3,4,5-trihydroxybenzoic acid, is produced by an acid or alkaline hydrolytic process. Gallic acid has been extensively studied for its potential as a food additive due to its antioxidant, antimicrobial, and anti-inflammatory properties [38]. It has been evaluated for its ability to inhibit lipid and anthocyanin oxidation, making it a promising additive in the preservation of food quality [39,40]. Furthermore, gallic acid has demonstrated biological potential in animal and cell models, particularly in the management of obesity and diabetes, by improving insulin sensitivity and renal function [41]. However, the effects of gallic acid in humans remain under investigation.

The gallic acid content in the IBPH determined in the present study (530.70 µg 100 g^−1^ dw) was higher than that reported in samples of pomegranate carpel membrane (404 µg 100 g^−1^ dw) and mesocarp (264 µg 100 g^−1^ dw) residues in the same fraction [42], as well as blackberry seed meal (345 µg 100 g^−1^ dw) [43]. However, the gallic acid content of this study was lower than that found in wine residues (18 000 µg 100 g^−1^ dw) extracted by alkaline hydrolysis [44].

On the other hand, biochanin A, the methylated form of the isoflavone genistein, was the most predominant flavonoid with 85.53% of the total flavonoids, followed by quercetin and quercitrin with 7.48 and 6.12%, respectively. In the present study, it is the first time that biochanin A present in a walnut product has been identified and quantified. Previously, only the isoflavone daidzein with traces of genistein was identified and quantified in *Juglans nigra* L. walnuts [45,46].

Biochanin A has been found primarily in red clover (*Trifolium pratense* L.), chickpea (*Cicer arietinum*), and other Fabaceae species like *Cassia fistula* and *Astragalus membranaceus* [47], with peanut skin also being a notable source [48]. It is recognized for its phytoestrogenic properties and potential therapeutic benefits, including anti-inflammatory effects. This natural compound has shown tyrosine kinase inhibition and antineoplastic and antidiabetic activities [49,50,51,52].

In the free phenolic fraction, gallic acid was the phenolic compound found in the highest concentration (3061.01 µg 100 g^−1^ dw) followed by caffeic acid (54.50 µg 100 g^−1^ dw) and 3,4-dihydroxybenzoic acid (43.32 µg 100 g^−1^ dw). These last two mentioned acids were only found in the free phenolic fraction. However, the flavonoid content in the free phenolic fraction represented only one-tenth of the phenolic acids.

In the esterified fraction of WMR, three phenolic acids were identified, of which *p*-coumaric and ferulic acids have a higher content compared to the free fraction, possibly indicating that a good part of these acids are linked by ester-like bonds to the matrix. This esterified fraction showed the highest significant content of biochanin A among all the fractions, representing almost 70% of the total found in WMR. This would indicate that this isoflavone is mostly bound by esterified bonds to sugar residues, amines, organic acids, lipids, and other phenolics [53]. It should be noted that only in this esterified fraction was the presence of flavone chrysin reported (8.67 µg 100 g^−1^ dw).

In the etherified fraction, two phenolic acids were identified (gallic and sinapic acids). Furthermore, sinapic acid was reported solely in this fraction, although in minimal amounts (2.11 µg 100 g^−1^ dw). In this fraction, the only flavonoid present in significant amounts was quercitrin, representing 18% of the total of this compound present in WMR. However, traces of quercetin and chrysin were also found.

In the insoluble phenolic fraction, the only phenolic compound identified of flavonoid type was quercetin in a significant amount (46% of the total of this compound). It is relevant to mention that the phenolic acids present in the IBPH represent more than 95% compared to the flavonoids present in this fraction. From the three phenolic acids identified in the IBPH, *p*-coumaric and ferulic acid are the ones found in the highest amount compared to the other fractions, representing 71.94 and 75.75%, respectively. This suggests that most of these acids are bound to the cell wall of the matrix.

It is important to highlight that, when considering the total content of phenolic acids and flavonoids quantified, only 14% are present in the IBPH, contrasting with the results obtained through the estimation of total phenolic compounds (TPCs). This could be due to interferences caused by the presence of other compounds with reducing capacity in WMR such as ascorbic acid (vitamin C), dehydroascorbic acid (oxidized form of vitamin C), transition metals, reducing amino acids (tryptophan and tyrosine), and reducing sugars. These non-phenolic compounds, soluble in the organic solvent used (methanol), may have reduced the Folin–Ciocalteu reagent, overestimating the TPC, which is a limitation of this spectrophotometric method. In contrast to this analysis technique, liquid chromatography (UPLC) coupled to mass spectrometry (MS) achieves not only the quantification but also the identification of the phenolic compounds present with high precision, resolution, efficiency, and reproducibility [54,55].

Some vegetable matrix residues such as peanut skin (92.07%) [11], araticum peel (76.16%) [56], and blueberry seed meal (62.74%) [43] present mostly soluble compounds. Likewise, the WMR presented 86% soluble compounds. The above could make the reuse of this residue more competitive since strategies for releasing insoluble-bound compounds, which would involve additional steps and expenses, would not be as necessary.

The literature data demonstrate that it is very difficult to predict which individual phenolic compound is responsible for the antioxidant potential in complex mixtures [57]. These authors addressed the antioxidant properties (FRAP and ORAC) of 10 phenolic acids, including gallic acid, *p*-coumaric acid, ferulic acid, and sinapic acids, which are present in the fractions recovered in the present study. By evaluating different mixtures (binary, ternary, quaternary, and quinary) at several different concentrations, it was possible to conclude that all effects (synergistic, antagonistic, or additive) depend on the combination and concentration, among other parameters. Therefore, the antioxidant properties of complex mixtures (e.g., phenolic extracts from plant by-products) are matrix dependent.

### 2.4. Cytotoxicity Against the Human Intestinal Cell Line (Caco-2)

The MTT assay measures cellular metabolic activity to indicate viability, proliferation, or cytotoxicity [58,59,60]. While multiple cell lines of human origin allow for cell viability assays, the Caco-2 cell line stands out [26,61,62,63]. It has been widely used to evaluate the effect of multiple compounds, extracts, and formulations on metabolism, viability, cell proliferation, and cytotoxicity [64,65,66,67,68,69]. This choice is based on the fact that Caco-2 cells represent, in a good way, one of the niches of interaction between compounds resulting from digestion and cells in charge of the uptake of bioactive compounds with potential use in humans. In addition, Caco-2 cells have been used to generate colonic epithelial barrier models to study the effects on intestinal permeability [70,71,72]. In these models, it is also possible to determine the presence of cytotoxic activity, alteration of cell integrity after chronic or acute exposure to food matrix extracts, and impaired redox balance [73,74].

The design used in this study sought to rule out the presence of cytotoxic compounds in a wide concentration range of the WMR free phenolic fraction as this was the one presenting the highest content of phenolics as evaluated by UPLC-ESI-MS/MS. An acute exposure (40 min) assay on a Caco-2 cell culture was considered. The MTT assay relates mainly mitochondrial reducing activity to cell viability and count. Notably, in acute assays (from minutes to a few hours), it is assumed that the cells need more time to divide, so the increase in assay values is attributed to an increase in reducing capacity rather than an increase in cell number. If this is the case, an increase in the reducing capacity value is equivalent to an increase in mitochondrial activity or another source of reducing power.

**Figure 1:**
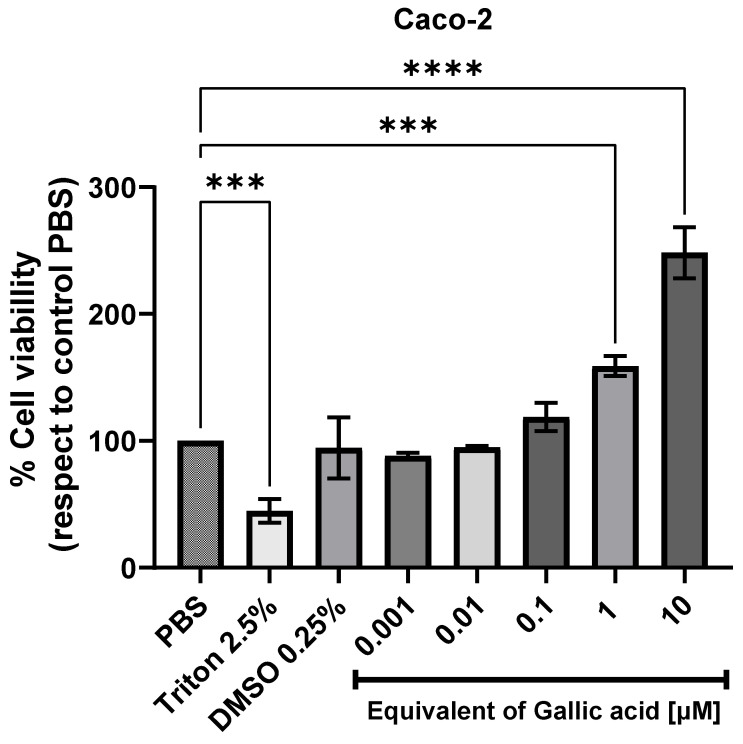
Effects of free phenolics of walnut milk residue (10, 1, 0.1, 0.01, and 0.001 µM equivalents of gallic acid as evaluated by UPLC-ESI-MS/MS) on the viability of human colorectal adenocarcinoma cell line (Caco-2) after exposure for 40 min. The assay was carried out using the MTT method, and the absorbance was monitored at a 570 nm wavelength. Data are expressed as mean ± SD (n = 3 independent experiments). Statistical comparison was performed using ordinary one-way (ANOVA) followed by Dunnett multiple comparisons test. **** and *** represents differences statically significant at *p* < 0.0001 and at *p* < 0.001, respectively with respect to the control (PBS) designated as 100% of viability.

The WMR extract positively stimulated cell metabolism at 1/100 and 1/10 dilutions, equivalent to 1 and 10 µM of gallic acid, respectively (Figure 1). Gallic acid at 10 µM (equivalent to the highest concentration tested in the free phenolic extract) was tested, demonstrating no detrimental effect (100% of cell viability, data not shown). Figure 1 also shows that no cytotoxic impact on Caco-2 cells was observed after 40 min compared to control conditions regardless of the dilution. On the contrary, a significant increase in cellular metabolic activities (reducing capacity) was observed at more concentrated conditions (10 and 1 µM equivalents of gallic acid). As mentioned earlier, this result indicated that the higher concentration of free phenolics in the WMR used in the assay increased the reducing properties, which could be attributed to the content of phenolics and lent support to the FRAP values (Section 2.2). Moreover, the lower concentration was not toxic, indicating that it was safe for Caco-2 cells.

To the best of our knowledge, a significant diversity of results in reports of cytotoxicity in Caco-2 cells underscores the complexity of extract compositions. These results vary depending on the type of extract and the exposure time. There are reports of extracts that affect viability in a dose-dependent manner; in some, an effect is only observed with high concentrations or prolonged exposure times, and in others, no effect is observed in the ranges studied. This variability highlights the need for further investigation and the potential impact of our findings on future research in this area.

## 3. Materials and Methods

### 3.1. Raw Material

The walnuts (Chandler variety) without a shell were obtained from a natural products store, harvested in autumn 2022 in the commune of Paine, Maipo province, Metropolitan Region of Santiago, Chile.

### 3.2. Solvents, Chemical Reagents, and Standards

Hexane, acetone, diethyl ether, ethyl acetate, methanol (reagent grade) hydrochloric acid, and Folin–Ciocalteu phenol reagent were purchased from Merck (Darmstadt, Germany). Sodium hydroxide, sodium carbonate (Na_2_CO_3_), 2,4,6-tri(2-pyridyl)-S-triazine (TPTZ), 6-hydroxy-2,5,7,8-tetramethylchromane-2-carboxylic acid (Trolox), sodium acetate trihydrate, ferric chloride (FeCl_3_), dibasic sodium phosphate (Na_2_HPO_4_), monobasic sodium phosphate (NaH_2_PO_4_), and fluorescein and 2,2’-azobis (2-amidinopropane) dihydrochloride (AAPH) were purchased from Sigma-Aldrich (St. Louis, MO, USA). All solvents and chemical reagents used were of analytical grade or higher. Phenolic standards, gallic acid (≥97.5%), *p*-coumaric acid (≥98.0%), caffeic acid (≥98%), ferulic acid (99%), sinapic acid (≥98%), biochanin A (≥96.5%), genistein (≥98%), quercetin (≥95%), chrysin (≥96.5%), and catechin (95%) were purchased from Sigma-Aldrich (St. Louis, MO, USA).

### 3.3. Walnut Processing Residues Preparation

The defatted walnut residue (DWR), used for comparative purposes, was obtained by liquefying the previously manually crushed walnuts with hexane solvent (solid/solvent, 1:5, *w*/*v*, 5 min) in a blender (Model Blender Base, Osterizer, Mexico) at room temperature three times [11,75]. The hexane-blended walnuts were vacuum filtered through #1 Whatman filter paper using a Buchner funnel. The DWR was dried in an oven for 24 h at 40 °C and then ground and stored at −20 °C.

WMR was prepared from 100 g of walnuts. They were soaked in distilled water for 8 h. Then, the walnuts were crushed in a homogenizer (MioMat Pro, China) at 42 °C for 12 min to obtain a homemade walnut milk. From the solid residue obtained after filtration of the homemade walnut milk (walnut milk residue, WMR), the lipid fraction was extracted in the same way as for obtaining the DWR, using hexane as solvent. The defatted WMR was subjected to an oven-drying process for 24 h at 40 °C and then milled and stored at −20 °C for further extractions.

### 3.4. Phenolic Fractions Extraction

**Figure 2:**
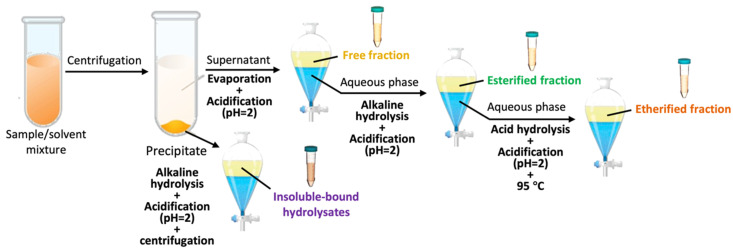
Extraction of soluble (free, esterified, and etherified) and insoluble-bound phenolic hydrolysates.

The soluble fraction from both residues (DWR and WMR) was used to obtain the free, esterified, and etherified fractions, while the insoluble fraction was used to obtain the IBPH, as briefly summarized in Figure 2.

#### 3.4.1. Soluble Phenolic Compounds Fractions Extraction

This extraction process was carried out following the procedure described by Ayoub et al. [43]. Soluble phenolics were extracted using acetone–water (1:1, *v*/*v*) as described by Rusu et al. [76]. Briefly, 3 g of the sample were extracted with 30 mL of the solvent (1:10, *w*/*v*). These samples were extracted to 30 °C × 20 min in a shaking bath (Model LSB-130S, LabTech, Gwangju-si, Republic of Korea). They were then centrifuged at 4000× *g* × 5 min (Model Z206A, HERMLE, Gosheim, Germany). After centrifugation, the supernatants were collected. This extraction process was repeated three times. The collected supernatants were used for the extraction of the fraction of free, esterified, and etherified soluble phenolics, and the solid residue was stored to subsequently extract the fraction of IBPH generated from the DWR and WMR. The organic solvent (acetone 50%) was removed under vacuum at 40 °C using a rotary evaporator (Model RE 100-Pro, California, USA) coupled to a pump (Vac V-500, BÜCHI, Flawil, Switzerland), obtaining an aqueous fraction of soluble phenolics. To obtain the fraction of free soluble phenolics, the aqueous fraction was pH adjusted to 2 and then washed with the same volume of the solvent mixture diethyl ether–ethyl acetate (1:1, *v*/*v*) five times. Between each wash, the sample was shaken and decanted, recovering the lower aqueous phase. The suspended organic phase was evaporated until a dry extract was obtained. This extract was dissolved in 5 mL of methanol and stored at freezing temperatures (−20 °C). Then, to obtain the fraction of esterified soluble phenolics, the aqueous phase was hydrolyzed with NaOH (2 M; 1:1, *v*/*v*) for 4 h at room temperature under a nitrogen atmosphere. Then, the hydrolyzed aqueous phase pH was adjusted to 2 with 6 M HCl, and the released phenolics were extracted with the mixture of diethyl ether–ethyl acetate (1:1, *v*/*v*). The solvent mixture was evaporated until a dry extract was obtained, which was suspended in 5 mL of methanol and subjected to freezing (−20 °C). The fraction of etherified soluble phenolics was obtained by previous pH adjustment to 2 with 6 M HCl from the aqueous phase collected from the extraction of the esterified phenol fraction. This acidified phase was incubated at 95 °C × 45 min in a thermoregulated bath with stirring. Then, the etherified phenolics were extracted with the mixture of diethyl ether–ethyl acetate (1:1, *v*/*v*). The solvent was evaporated until a dry extract was obtained, which was dissolved in 5 mL of methanol and stored at freezing temperatures (−20 °C) for further analysis.

#### 3.4.2. Insoluble-Bound Phenolic Hydrolysates

To obtain the IBPH, the solid residue obtained from the extraction of the soluble phenolics was dissolved in 2 M NaOH (20 mL g^−1^ sample) and stirred for 4 h at room temperature under a nitrogen atmosphere. The mixture was adjusted to pH 2 with 6 M HCl. Then, phenolics released from their insoluble-bound form (IBPH) were extracted with a mixture of diethyl ether–ethyl acetate (1:1, *v*/*v*) five times, being subjected to centrifugation to facilitate the separation of the phases (organic and aqueous). The solvent mixture was evaporated under vacuum until a dry extract was obtained, which was suspended in a determined volume of methanol and stored at freezing temperatures (−20 °C) for further analysis.

### 3.5. Total Phenolic Compounds (TPCs)

Total phenolics were analyzed according to the methodology described by Swain and Hillis [77] with certain modifications. Briefly, 450 µL of distilled water was mixed with 400 µL of sodium carbonate (20%, *w*/*v*) in a test tube. Then, the extract diluted in methanol (150 µL) was added followed by Folin–Ciocalteu phenol reagent (2000 µL). For blank preparation, 150 µL of methanol was added instead of the extract. These preparations were kept at 37 °C × 30 min. Then, the absorbance was measured at 765 nm in a spectrophotometer (N4S, China). The content of total phenolic compounds was expressed as mg of gallic acid equivalent (GAE) per 100 g of sample in dry weight.

### 3.6. Ferric Reducing Antioxidant Power (FRAP)

The reducing power of the extracts was determined following the procedure described in the literature [53,78]. FRAP reagent was prepared by mixing 50 mL of acetate buffer solution (pH 3.6), 5 mL of TPTZ solution diluted in HCl (40 mM), and 5 mL of ferric chloride solution (FeCl_3_). Aliquots of 300 µL of sample were mixed with 2700 µL of FRAP reagent in a test tube. The samples were then incubated in the dark for 30 min at room temperature. Absorbance readings were performed at 593 nm in a UV-vis spectrophotometer (N4S, China) in triplicate for each sample. The calibration curve was constructed with the Trolox standard at concentrations from 25 to 200 µM. The results were expressed as µmol of Trolox equivalents per gram of sample.

### 3.7. Oxygen Radical Absorbance Capacity (ORAC)

The ORAC assay was performed following the methodology described by de Camargo et al. [79] with certain modifications. Aliquots of 175 µL of phosphate buffer (pH 7.4), 30 µL of fluorescein, and 20 µL of the sample were mixed in a 96-well microplate. The mixtures were incubated for 20 min at 37 °C inside the Infinite Pro 200 microplate reader (TECAN, Männedorf, Switzerland). Then, 25 µL of AAPH was added to each well. Fluorescence readings were performed every 2 min for 140 min with excitation and emission wavelength of 480 nm and 510 nm, respectively. The Trolox standard was employed at concentrations from 2.5 µM to 10 µM. The results were expressed as µmol of Trolox equivalents per gram of sample.

### 3.8. UPLC-ESI-MS/MS Analysis from WMR

The ultra-performance liquid chromatography–electrospray tandem mass spectrometry coupled to tandem mass spectrometry (MS/MS) was carried out following the methodology described by de Camargo et al. [53], with minor modification, using a 4500 triple quadrupole mass spectrometer equipped with an ionization electrospray Turbo V attached to an Eksigent Ekspert Ultra 100 liquid chromatograph with an autosampler system (AB/Sciex, Concord, ON, Canada). The electrospray was employed in negative mode considering the following parameters: curtain gas = 30 psi, collision gas = 10 psi, ion spray voltage = −4500 V, temperature = 650 °C, ion source gas 1 = 50 psi, ion source gas 2 = 50 psi, and input potential = −10 V. The chromatographic separation was performed considering a gradient elution with two mobile phases: (A) 0.1% formic acid and (B) methanol. The gradient was carried out as follows: 0.1 min, 5% B; 1–12 min, 5–50% B; 12–13 min 50–50% B; 13–14 min, 50–5% B; and 14–15 min, 5% B. The injection volume was 10 µL with a flow rate of 0.5 mL/min and an end-capped column (LiChrospher 100 RP-18; 125 mm, 4 mm, 5 µm; Merck, Darmstadt, Germany) maintained at 50 °C. Commercial standards were used to construct calibration curves for quantification of the compounds present in the samples. The identification of phenolic compounds was carried out using multiple reaction monitoring (MRM) and authentic standards by UPLC-ESI-MS/MS (Table 3) according to previous studies by our research group [53,80]. Limits of detection (LOD), limit of quantification (LOQ), and the r^2^ of the plotted graphs were as follows: gallic acid (LOD = 41 ppb, LOQ = 124 ppb, and r^2^ = 0.9988); *p*-coumaric acid (LOD = 124 ppb, LOQ = 377 pbb, and r^2^ = 0.9911); caffeic acid (LOD = 142 ppb, LOQ = 430 ppb, and r^2^ = 0.9976); ferulic acid (LOD = 110 ppb, LOQ = 334 ppb, and r^2^ = 0.9944); sinapic acid (LOD = 120 ppb, LOQ = 364 ppb, and r^2^ = 0.9984); genistein (LOD = 105 ppb, LOQ = 319 ppb, and r^2^ = 0.9908); quercetin (LOD = 67 ppb, LOQ = 203 ppb, and r^2^ = 0.9969); catechin (LOD = 49 ppb, LOQ = 150 ppb, and r^2^ = 0.9997).

### 3.9. Cell Culture

The human colorectal adenocarcinoma cell line Caco-2 (HTB-37TM) cells were acquired from the American Type Culture Collection (ATCC R, Manassas, VA, USA). Cell lines were cultured at 37 °C in Dulbecco’s modified Eagle’s medium (DMEM) high glucose (Thermo Fisher Scientific, Waltham, MA, USA), supplemented with 10% (*v*/*v*) fetal bovine serum (FBS) (Biological Industries, Beit HaEmek, Israel), and 1% of antibiotic (penicillin and streptomycin) in 75 cm^2^ flasks and a humidified atmosphere with 5% CO_2_.

### 3.10. 3-[4,5-Dimethylthiazol-2-yl]-2,5-Diphenyltetrazolium Bromide (MTT) Cell Viability Assay

An MTT (3-(4,5-dimethylthiazol-2-yl)-2,5-diphenyl-2H-tetrazolium bromide) assay was conducted to indirectly assess the effect of walnut extract on Caco-2 cell viability. Initially, cells (5 × 10^4^) were seeded at 200 µL per well in a 96-well microplate for 24 h. Before the experiments, cells were washed two times with PBS 1X and then exposed for 40 min to different dilutions of phenolic extract (10, 1, 0.1, 0.01, and 0.001 µM equivalents of gallic acid as evaluated by UPLC-ESI-MS/MS) dissolved in PBS-DMSO, as indicated above. PBS-DMSO 0.25% and PBS-Triton X-100 at 1% (*v*/*v*) were applied as a vehicle and positive control of cell death, respectively. After the exposition, when MTT is reduced in living cells by the NAD(P) H-dependent oxidoreductase enzymes, the central tetrazole ring breaks, creating the purple, water-insoluble chemical known as formazan. Then, formazan crystals were solubilized by adding 100 μL of DMSO–isopropanol (3:2), and absorbance (570 nm) was determined using a multi-plate reader (Tecan Infinite F200 Microplate Reader, New York, NY, USA) as described [58]. Results from three independent experiments are expressed as the average percentage of control (PBS) designated as 100% of viability. Vehicle control (PBS-DMSO used to dissolve the extract) was not statistically different.

### 3.11. Statistical Analysis

All analyses were performed in triplicate for the respective standards and samples. The results for total phenol content (TPC), UPLC-ESI-MS/MS, reducing power (FRAP), and antioxidant potential (ORAC) were analyzed by an analysis of variance (ANOVA) and Tukey test (*p* < 0.05) using Statgraphics Centurion XV.I software to determine the significant differences between samples. In addition, the results obtained by ORAC and MTT were evaluated using GraphPad Prism 5 software.

## 4. Conclusions

In summary, it was shown that 44% *w*/*w* of the phenolics present in the walnut were not extracted during the production of walnut milk and remained in the respective residue (WMR). WMR contains mainly soluble phenolic antioxidants, mostly in the free form. Gallic acid was the most representative phenolic compound found in WMR. Moreover, biochanin A isoflavone was identified and quantified for the first time in a product from walnuts in significant amounts mainly in the esterified phenolic fraction. No detrimental cytotoxic impact on Caco-2 cells was found. Therefore, WMR could be considered as a potential source of natural antioxidants contributing to the nutraceutical and food industry in the framework of sustainable processes.

## Figures and Tables

**Table 1:** Total phenolic content (TPC), reducing power (FRAP), and antioxidant potential (ORAC) of the soluble phenolics (free, esterified, and etherified) and insoluble-bound phenolic hydrolysates (IBPHs) fractions of defatted walnut residue (DWR) and walnut milk residue (WMR).

Sample	Free	Esterified	Etherified	IBPH	Total
Total phenolic content (mg GAE 100 g^−1^ dw)					
DWR	384.51 ± 9.8 ^Aa^	141.50 ± 2.2 ^Ab^	104.15 ± 2.9 ^Ac^	373.48 ± 5.0 ^Aa^	1003.64
WMR	124.45 ± 3.5 ^Ba^	78.30 ± 0.9 ^Bb^	72.55 ± 0.7 ^Bb^	167.95 ± 3.3 ^Bc^	443.25
Ferric Reducing Antioxidant Power_FRAP (µmol TE g^−1^ dw)					
DWR	887.56 ± 37.5 ^Aa^	79.70 ± 5.9 ^Ab^	79.48 ± 5.3 ^Ab^	167.07 ± 9.4 ^Ac^	1213.81
WMR	109.64 ± 9.5 ^Ba^	19.52 ± 1.4 ^Bb^	8.97 ± 0.9 ^Bb^	57.94 ± 0.9 ^Bc^	196.07
Oxygen radical absorbance capacity_ORAC (µmol TE g^−1^ dw)					
DWR	36.80 ± 2.0 ^Ab^	20.24 ± 0.4 ^Ad^	24.70 ± 0.0 ^Ac^	50.47 ± 1.8 ^Aa^	132.21
WMR	15.52 ± 0.1 ^Bb^	13.84 ± 0.1 ^Bc^	16.05 ± 0.8 ^Bb^	20.84 ± 0.0 ^Ba^	66.25

Data represent values of the mean ± standard deviation (n = 3). Means followed by different capital letters within a part of a column show significant differences between the two samples (*p* < 0.05). Means followed by different lowercase letters within a row show significant differences between phenol fractions (*p* < 0.05). Abbreviations: GAE, gallic acid equivalents; TE, Trolox equivalents; d.w., dry weight. DWR phenolic extraction yield; free: 0.38, esterified: 0.14, etherified: 0.10, and IBPH: 0.37% (*w*/*w*). WMR phenolic extraction yield; free: 0.12, esterified: 0.08, etherified: 0.07, and IBPH: 0.17% (*w*/*w*).

**Table 2:** Phenolic compounds (phenolic acids and flavonoids) of the soluble (free, esterified, and etherified) and insoluble-bound phenolic hydrolysates (IBPHs) of walnut milk residue (µg 100 g^−1^).

Compound	Free	Esterified	Etherified	IBPH	Total
Phenolic acids					
Gallic acid	3061.01 ± 19.71 ^Aa^	88.87 ± 3.83 ^Bb^	88.10 ± 0.87 ^Ab^	530.70 ± 3.89 ^Bc^	3768.68
*p*-Coumaric acid	18.15 ± 1.97 ^Ba^	25.43 ± 0.08 ^Ca^	tr	111.73 ± 5.65 ^Cb^	155.31
Caffeic acid	54.50 ± 0.63 ^C^	tr	nd	tr	54.50
Ferulic acid	5.70 ± 0.44 ^Ba^	7.38 ± 0.75 ^Aa^	tr	40.83 ± 3.14 ^Ab^	53.90
3,4-Dihydroxybenzoic acid *	43.32 ± 0.95 ^C^	nd	nd	nd	43.32
Sinapic acid	tr	tr	2.11 ± 0.15 ^B^	tr	2.11
Flavonoids					
Biochanin A *	259.17 ± 20.78 ^Aa^	593.75 ± 1.50 ^Bb^	nd	nd	852.92
Quercetin	40.30 ± 1.37 ^Ba^	tr	tr	34.26 ± 0.64 ^b^	74.56
Quercitrin *	37.49 ± 1.18 ^Ba^	12.53 ± 0.15 ^Cb^	11.03 ± 0.69 ^b^	tr	61.05
Chrysin *	tr	8.67 ± 0.36 ^A^	tr	tr	8.67

tr, traces; nd, not detected. * 3,4-Dihydroxybenzoic acid, biochanin A, quercitrin, and chrysin were quantified as equivalents of gallic acid, genistein, quercetin, and catechin, respectively. Means followed by different capital letters within a part of a column show significant differences (*p* < 0.05). Means followed by different lowercase letters within a row show significant differences between phenol fractions for each quantified phenolic compound (*p* < 0.05).

**Table 3:** Phenolic compounds of walnut milk residue identified by UPLC–ESI-MS/MS.

Compound	MRM * Transition 1	DP	CE	CXP	MRM Transition 2	DP	CE	CXP
Gallic acid	168.9 ** > 124.9 ***	−70	−18	−7	168.9 ** > 78.9 ***	−70	−18	−7
*p*-Coumaric acid	162.9 ** > 119.0 ***	−70	−20	−5	162.9 ** > 119.0 ***	−70	−38	−25
Caffeic acid	178.9 ** > 135.0 ***	−70	−20	−5	178.9 ** > 133.9 ***	−70	−32	−7
Ferulic acid	193.0 ** > 134.0 ***	−55	−20	−7	193.0 ** > 177.9 ***	−55	−16	−15
Sinapic acid	223.0 ** > 207.9 ***	−75	−18	−7	223.0 ** > 148.8 ***	−75	−26	−13
3,4-Dihydroxybenzoic acid	152.9 ** > 107.9 ***	−50	−26	−3	152.9 ** > 108.9 ***	−50	−16	−3
Biochanin A	282.9 ** > 267.9 ***	−80	−32	−5	282.9 ** > 211.1 ***	−80	−46	−5
Quercetin	301.0 ** > 150.9 ***	−15	−28	−13	301.0 ** > 178.8 ***	−15	−24	−11
Quercitrin	447.0 ** > 299.9 ***	−105	−32	−13	447.0 ** > 299.9 ***	−105	−30	−7
Chrysin	253.0 ** > 208.9 ***	−130	−16	−7	253.0 ** > 143.0 ***	−130	−34	−3

* MRM, [multiple reaction monitoring]; ** Precursor ions *m*/*z*; *** Product ions *m*/*z*; CE, [collision energy]; CXP, [collision cell exit potential]; DP, [de-clustering potential].

## Data Availability

The data used to support the findings of this study can be made available by the corresponding author upon request.
